# Evolutionary Genomics of a Temperate Bacteriophage in an Obligate Intracellular Bacteria (*Wolbachia*)

**DOI:** 10.1371/journal.pone.0024984

**Published:** 2011-09-14

**Authors:** Bethany N. Kent, Lisa J. Funkhouser, Shefali Setia, Seth R. Bordenstein

**Affiliations:** Department of Biological Sciences, Vanderbilt University, Nashville, Tennessee, United States of America; University of Poitiers, France

## Abstract

Genome evolution of bacteria is usually influenced by ecology, such that bacteria with a free-living stage have large genomes and high rates of horizontal gene transfer, while obligate intracellular bacteria have small genomes with typically low amounts of gene exchange. However, recent studies indicate that obligate intracellular species that host-switch frequently harbor agents of horizontal transfer such as mobile elements. For example, the temperate double-stranded DNA bacteriophage WO in *Wolbachia* persistently transfers between bacterial coinfections in the same host. Here we show that despite the phage's rampant mobility between coinfections, the prophage's genome displays features of constraint related to its intracellular niche. First, there is always at least one intact prophage WO and usually several degenerate, independently-acquired WO prophages in each *Wolbachia* genome. Second, while the prophage genomes are modular in composition with genes of similar function grouping together, the modules are generally not interchangeable with other unrelated phages and thus do not evolve by the Modular Theory. Third, there is an unusual core genome that strictly consists of head and baseplate genes; other gene modules are frequently deleted. Fourth, the prophage recombinases are diverse and there is no conserved integration sequence. Finally, the molecular evolutionary forces acting on prophage WO are point mutation, intragenic recombination, deletion, and purifying selection. Taken together, these analyses indicate that while lateral transfer of phage WO is pervasive between *Wolbachia* with occasional new gene uptake, constraints of the intracellular niche obstruct extensive mixture between WO and the global phage population. Although the Modular Theory has long been considered the paradigm of temperate bacteriophage evolution in free-living bacteria, it appears irrelevant in phages of obligate intracellular bacteria.

## Introduction

Bacteriophages, viruses that infect bacteria, play a major role in bacterial genome evolution and ecology through their global abundance [1] and their ability to laterally transfer their genomes between bacteria [2], [3], [4]. The most common bacterial viruses, the double-stranded (ds)DNA tailed phages, outnumber prokaryotic cells by 10-fold in environmental samples [5] and are responsible for the majority of intraspecific genome diversification in bacteria [6], [7], [8]. This diversification is due in part to bacteriophages triggering genomic rearrangement in their host bacteria and transmitting new genetic material both within and sometimes between different bacterial species. A striking example of bacteriophage diversity is found in *Mycobacterium smegmatis*, where eighty different dsDNA tailed phages from 21 different viral clusters are present [9], [10], [11], [12]. Bacteriophages are also known to alter bacterial cell biology by facilitating the transfer of virulence factors such as superantigens, extracellular toxins, effector proteins that modulate host-cell invasion, and host-cell adhesion factors [13].

The Modular Theory of dsDNA phages, as originally proposed by Botstein (1980), asserts that phage genomes consist of conserved clusters of functionally-related genes (i.e. modules) that can be interchanged by horizontal transfer among a large common phage gene pool [14], [15]. These phage modules are composed of contiguous sets of genes involved in a similar function, such as head assembly, tail formation, or regulation of the lysis and lysogeny cycles. While the genome of the phage is the total composite of the phage's DNA, the Modular Theory asserts that phage evolution primarily acts at the level of gene modules due to promiscuous module exchange between unrelated phages, where essentially one module is replaced with another that has the same general biological function. Comparative approaches suggest modularity and mosaicism are major evolutionary hallmarks of dsDNA phages. However, generalizing the principles to all dsDNA phages will require an expanded analysis of phage genomics in diverse ecological ranges [16]. In this regard, obligate intracellular bacteria, which live and replicate within the cytosol of host cells, are an ideal test of the Modular Theory since the intracellular niche may pose ecological restraints on exposure to novel phage gene pools.

The genome sequences of obligate intracellular bacteria have brought a renewed interest in mobile element evolution in bacteria prone to genome reduction [17], [18], [19], [20], [21], [22], [23], [24], [25], [26], [27], [28], [29], [30]. Comparative analyses of multiple genomes of obligate intracellular species demonstrate that although these bacteria have tiny genomes, their ecological range correlates with mobile element abundance. Specifically, species that host-switch tend to harbor significantly more mobile elements than those species that transmit vertically through the maternal line [31], [32]. Of the mobile elements studied in obligate intracellular bacteria, temperate bacteriophages are noteworthy for their ability to spread intercellularly [28], [33], [34], [35] and diversify the host bacterial genome [36].

At least three arguments suggest phages from host-restricted bacteria may not evolve by the Modular Theory. First, point mutations can be the major cause of phage diversification [37] across a core genome that is recalcitrant to lateral gene transfer. Second, some phage types, such as the T4-like phages, show a mixed genomic structure involving both modular exchanges and a conserved core genome [38]. Third, and perhaps most importantly, constraints on phage evolution in a restricted intracellular niche could suppress recombination with novel gene pools and lead to a preponderance of single nucleotide mutations and deletions.

Here we test for the first time whether the Modular Theory governs the genome evolution of double-stranded DNA phages in an obligate intracellular genus (*Wolbachia*). The WO bacteriophages are an ideal system to discern the evolutionary forces that are shaping phage genome and protein evolution in obligate intracellular bacteria. *Wolbachia* are predicted to infect two out of three arthropod species globally [39], [40], [41] in addition to 90% of filarial nematode species [42]. Extrapolation of the infection frequency using estimates of the number of arthropod species makes this bacterium one of the most infectious microbes on the planet. *Wolbachia* are transmitted both vertically within species and horizontally between species, which promotes a higher exposure rate to novel gene pools. For this study, we selected sequences from three complete *Wolbachia* genomes (*w*Mel, *w*Pip, and *w*Ri) containing WO prophages [29], [30], [43] and complete prophage sequences from two additional *Wolbachia* (*w*CauB and *w*VitA) [28], [44]. These five *Wolbachia* induce cytoplasmic incompatibility, a reproductive modification that typically results in embryonic lethality between crosses of infected males and uninfected females. Each fully sequenced *Wolbachia* genome contains two to five prophage WO haplotypes, demonstrating that phage diversity is common within *Wolbachia* genomes. Molecular surveys have placed bacteriophage WO in 89% of *Wolbachia* from the A and B phylogenetic supergroups that infect arthropods [45], [46]. Thus, the abundance of phage WO and its ability to rampantly transfer between *Wolbachia* coinfections [28], [33], [35], [45] in one of the most prevalent bacterial infections in animals demonstrates its potential impact on bacterial symbiont diversity and host-symbiont interactions.

To determine the molecular evolutionary forces shaping prophage WO genomes, we addressed four interconnected questions. (i) First, does the Modular Theory explain the genetic changes in WO genomes or do point mutations provide most of the genetic diversity? (ii) Second, does the obligate intracellular niche constrain the acquisition of new genes and/or modules in WO prophages? (iii) Third, is the WO integration site and mechanism conserved in *Wolbachia*? We explore WO integration by comparing the recombinases encoded in each WO type and the areas of the host *Wolbachia* genome surrounding the integrated prophages. (iv) Finally, what is the relative strength of selection and recombination on prophage WO protein evolution across the functional modules of the genome?

## Results

### I. Does the Modular Theory explain the evolution of prophage WO genomes?

**Figure 1:**
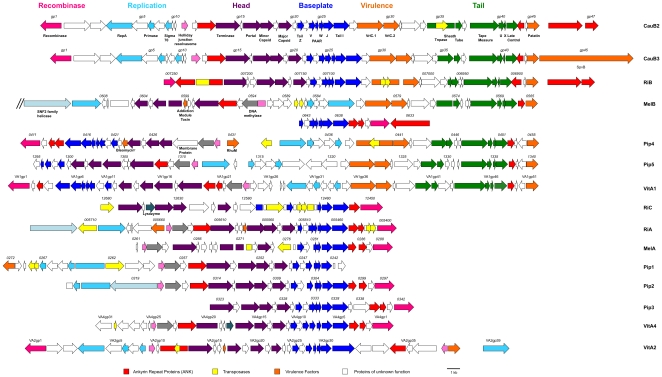
Prophage WO genomes are modular. A schematic of gene synteny across the prophage WO genomes is depicted. Complete WO prophage sequences are available with the exception of *w*Pip2 and *w*Pip3, as the *w*Pip genome sequence was artificially connected between genes within these two prophages [29]. These two prophages are treated as separate and complete. The two prophages from *w*CauB have been shown to be excisable by the mapping of their *att* sites [44] in conjunction with visualizing phage particles [44], [47]. Inverse PCR and sequencing analysis showed that WOCauB2 is conjoined between the integrase B2gp1 and the ankyrin repeat protein B2gp47, and WOCauB3 is conjoined between the integrase B3gp1 and the putative SpvB family toxin B3gp45 and hypothetical protein encoding B3gp46 genes [44]. WO haplotypes from *w*Mel [46], *w*Pip [49], and *w*VitA [48] are presumed excisable due to observations of lytic phage particles in each system. Genes are colored based on functional type and homology. Bright pink: integrase/recombinase; Red: Ankyrin-repeat protein; Turquoise: Replication module; Purple: Head module; Blue: Baseplate module; Orange: Putative virulence factors; Green: Tail module; Yellow: Transposases; Light pink: Holliday junction resolvasome/endonuclease; Grey: DNA methylase, Light teal: SNF2 helicase, Dark teal: lysozyme. The numbers above genes refer to the locus tag of that gene in the published genome.

Comparative sequence analyses of 16 prophage WO genomes from *Wolbachia* strains that induce cytoplasmic incompatibility (Table 1) specify the largest WO prophages, WOCauB2 and WOCauB3, as 43.2 kb (46 genes) and 45.2 kb (47 genes), respectively. We define each prophage as a contiguous set of phage-related genes and each haplotype as a genetic variant of the prophage WO family. There are six haplotypes that are capable of forming virions, including WOCauB2 and WOCauB3 [47], WOVitA1 [28], [48], and at least one haplotype each from *Wolbachia* infecting *Culex pipiens* [49], *Drosophila simulans*, and *D. melanogaster* [50]. For the analyses below, the prophage region cutoffs for each haplotype are estimated according to the terminal genes of the WOCauB2 and WOCauB3 reference genomes. The first and main observation from these comparisons (Figure 1) is that the WO haplotypes do not exemplify one of the two patterns consistent with the Modular Theory [14]. While the genomes are modular in nature, there is no evidence of promiscuous exchange of functional modules between unrelated phages. The closest sequence relatives of all gene modules in WO are from other WO haplotypes based on nucleotide and protein BLAST searches. Thus, the recent, genetic changes of prophage WO within the niche of *Wolbachia* principally arose from descent with modification. However, the ancestral gene modules of WO were previously annotated to be from diverse phages [44],

**Table 1:** Prophage strains used in this study.

Prophage	Host insect	Common Name	*Wp* group	*Wp* strain	Rp	ORFs	Head/Bp	Vir./Tail	Rec.	Rep.	Unchar.	Ref
WOCauB2	*Cadra cautella*	moth	B	*w*CauB	CI	B2gp1-gp47	gp11-gp27	gp28-gp47	gp1-gp3	gp4-gp10	NA	[44]
WOCauB3						B2gp1-gp46	gp12-gp28	gp29-gp46	gp1-gp3	gp4-gp11	NA	
WOPip1	*Culex pipiens*	mosquito	B	*w*Pip	CI	WPa_0242-0272	WPa_0242-0261	NA	NA	WPa_0262-0270	WPa_0271-0272	[29]
WOPip2						WPa_0297-0322	WPa_0301-0318	NA	NA	WPa_0319-0322	NA	
WOPip3						WPa_0323-0342	WPa_0323-0336	NA	WPa_0337-0342	NA	NA	
WOPip4						WPa_0411-0455	WPa_0415-0430	WPa_0438-0455	WPa_0411-0414	WPa_0433-0437	WPa_0431-0432	
WOPip5						WPa_1294-1340	WPa_1294-1311	WPa_1321-1340	NA	WPa_1312-1318	WPa_1319-1320	
WOMelA	*Drosophila melanogaster*	fruit fly	A	*w*Mel	CI	WD_0261-0288	WD_0261-0284	NA	WD0285-0288	NA	NA	[30]
WOMelB1						WD_0565-0610	WD_0593-0605	WD_0581-0565	NA	WD_0582-0592	WD_0606-0610	
WOMelB2						WD_0634-0644	WD_0638-0644	NA	WD_0634-0637	NA	NA	
WORiA-1	*Drosophila simulans* Ri.	fruit fly	A	*w*Ri	CI	wRi_005400-005720	wRi_005460-005650	NA	wRi_005400-005450	NA	wRi_005660-005720	[43]
WORiA-2						wRi_010060-010380	wRi_010120-010310	NA	wRi_010060-010110	NA	wRi_010320-010380	
WORiB						wRi_006880-007250	wRi_p07230-007070	wRi_007060-006880	wRi_p07240-007250	NA	NA	
WORiC						wRi_012450-012670	wRi_012670-012470	NA	wRi_012450-012460	NA	NA	
WOVitA1	*Nasonia vitripennis*	jewel wasp	A	*w*VitA	CI	VA1gp1-gp51	VA1gp5-gp23	VA1gp35-gp51	VA1gp1-gp4	VA1gp27-gp34	VA1gp24-gp26	[28]
WOVitA2						VA2gp1-gp39	VA2gp9-gp31	NA	VA2gp1-gp3	VA2gp4-gp8	VA2gp32-gp39	
WOVitA4						VA4gp1-gp31	VA4gp5-gp27	NA	VA4gp1-gp4	NA	VA4gp28-gp31	

*Wp-Wolbachia pipientis*; Rp- reproductive parasitism; CI – cytoplasmic incompatibility; NA – not applicable; Bp – Baseplate; Vir. – Virulence; Rec. – Recombinase; Rep. – Replication; Unchar. – Uncharacterized. WOVitA3 was not analyzed because the initial identification via PCR product published in [45] was found to be a chimera. For the purposes of this study, WOMelB1 and WOMelB2 are combined into one haplotype, WOMelB; see text for justification. Similarly, WORiA-1 and WOiRA-2 are analyzed as one haplotype, WORiA, since they are identical copies.

Second, each *Wolbachia* strain has at least one complete prophage with head, baseplate, virulence, and tail modules in addition to one or more partial prophages (Figure 1). We classify partial prophages as genomes that lack one of these modules; they are unlikely to be active by themselves as they are generally missing tail genes that are required for adsorption and infection. However, an intact copy of each known structural gene in a *Wolbachia* genome could allow for bacteriophage protein “commandeering” where the prophages that lack the tail module could use proteins encoded by the other functional haplotype within the genome to complete their assembly and movement. Alternatively, these partial prophages may form virions that are tailless or they do not form virions at all.

The presence of partial prophage sequences can be explained by three possible scenarios: (i) recurrent infections by new WO types followed by degeneration, (ii) duplications of the resident WO haplotype(s) by errors in replication or recombination, followed by degeneration of one of the copies, or (iii) a combination of the two scenarios. To distinguish these alternatives, we compared the average nucleotide identity of seven WO prophage genes within each *Wolbachia* to that between different *Wolbachia* (Table S1). If the haplotypes arose by duplication within a *Wolbachia* genome, then we expect to observe higher prophage sequence homology within a *Wolbachia* genome than between them. Six of the genes selected for this analysis are homologs of WOCauB2 genes gp17, gp18, gp19, gp21, gp22, and gp23; they occur in all of the prophages. The seventh gene is a homolog of WOCauB2 gp15 that is absent only in WOPip4. The average nucleotide identity of these prophage genes within a *Wolbachia* genome ranged from 53.4% in *w*CauB (gp23) to 99.1% in *w*Pip (gp15) (Table S1). The genes analyzed from most WO phages never had the highest level of nucleotide identity with another prophage in the same *Wolbachia* genome. For example, WOMelB and WORiA-1 and WORiA-2 (identical copies, hereafter referred to as WORiA) are more closely related to each other (99.9% identical) than to the other prophages within their *Wolbachia* genomes (75.6% in *w*Mel and 80% in *w*Ri). One exception is strain *w*Pip from *Culex pipiens*, where prophage WO genes are more likely to have their closest homolog in the same genome. The genes in WOPip1, WOPip2, and WOPip3 are most closely related to each other, with an average of 93.5% similarity, when compared to other WO prophages (MWU, two-tailed, p<0.001). For five of the seven genes and four out of six genes, respectively, WOPip5 and WOPip4 are most closely related to another *w*Pip prophage. Therefore, with the exception of *w*Pip that appears prone to within-genome WO duplications, independent acquisition of new WO haplotypes explains the variation within a single *Wolbachia* genome.

A third observation of prophage WO genomes is that while the genes are syntenous within modules, the position of the modules within the prophages is highly variable. For instance, Figure 1 shows that in WOCauB2, WOCauB3, and WORiB, the head, baseplate, and tail genes are oriented in the same direction. However, in WOPip4, WOPip5, and WOVitA1, the head and baseplate modules are inverted compared to the tail module. In WOMelB, the head and tail module (denoted WOMelB1) are contiguous but inverted from each other, while the presumed baseplate module and the recombinase (WOMelB2) that belong to this prophage are located 34.7 kb downstream. These two fragments of the prophage were putatively conjoined at one point because they are proximate to each other. The insertion between them is derived from a lateral transfer event with a *Rickettsia* plasmid [36], and the two prophage fragments complement each other exactly to make an intact prophage. Interestingly, despite the orientation of the other modules in the genome, the recombinase gene is always positioned so that the 3′ end is adjacent to the flanking region of the prophage (Figure 1), which typically contains an ankyrin repeat family protein.

WO is temperate and should therefore have identifiable endolysin genes. Surprisingly, the prophages do not contain a conserved endolysin, despite electron micrograph evidence that phage WO can lyse *Wolbachia* [35], [48]. No holins and only two lysozymes (in WOVitA4 and WORiC) were identified in the WO prophages. Therefore, the proteins encoding the lysin by which WO exits the bacterial cell may be novel. The patatin-like phospholipase encoded on the terminal portion of the tail module could be involved in cell exit or entry of the bacteriophage. Likewise, other than the integrase, proteins that comprise a typical lysogeny module, such as transcriptional regulators, are unidentified.

A RepA-family helicase, a primase, and a sigma-70 subunit that may direct the bacterial RNA polymerase to these genes for initiation of DNA strand synthesis are present in the predicted replication module. At least one of the genes is encoded in ten of the sixteen WO prophages, but only six WO genomes encode all three genes. Two other genes with a predicted function, a Holliday junction resolvasome/endonuclease and a DNA methylase, are present that could be involved in DNA replication and packaging. These two genes are adjacent to each other and lie between the replication and head modules in the ten prophages for which they are both present. The endonuclease could assist DNA packaging of mature phage heads by cleaving branched DNA structures of replicated phage DNA. The methylases may modify the packaged phage DNA such that it becomes resistant to bacterial restriction systems. The endonuclease is present in three additional prophages in which the methylase is absent. Interestingly, the endonuclease and methylase genes are oriented so that they appear to be part of the head/baseplate modules and not the replication module. The endonuclease is likely to degrade either bacterial DNA to inhibit the host during WO's lytic cycle or superinfecting phage DNA. In three prophages (WOMelB, WORiA-1, and WORiA-2), a second *repA* gene is present and adjacent to a SNF2-family helicase. In WOPip2, the SNF2 helicase is part of the replication module that also contains a single *repA* and the sigma-70 subunit.

### II. Does the obligate intracellular niche constrain the acquisition of new genes in WO phages?

**Figure 2:**
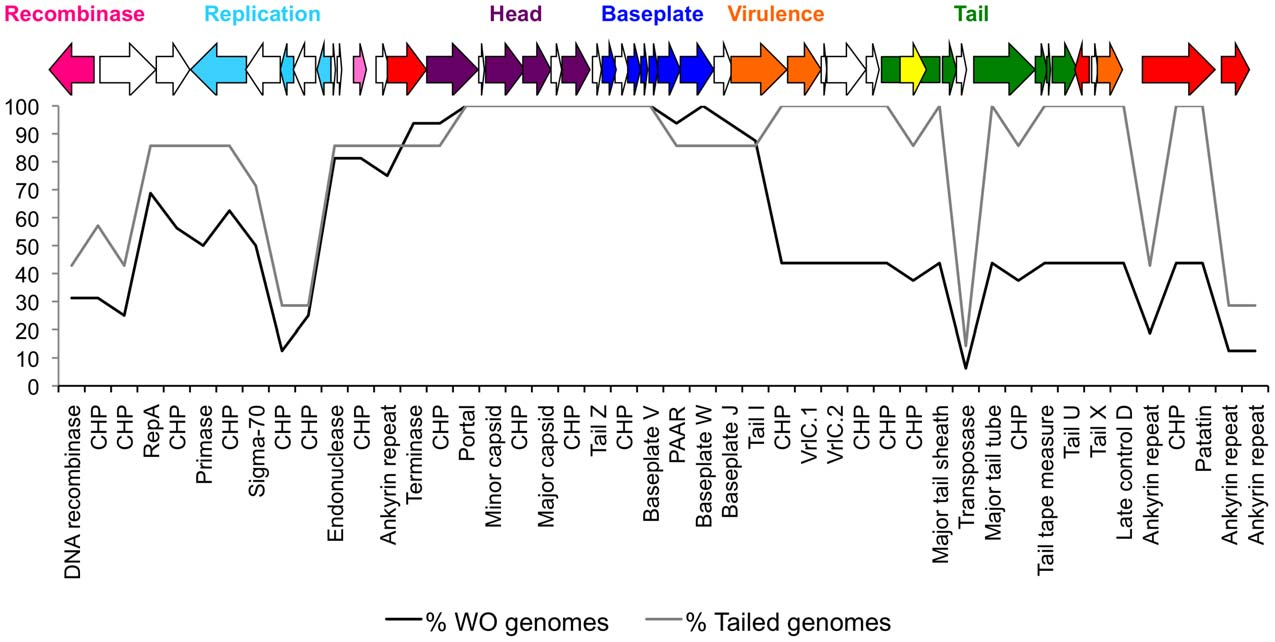
The core genome of prophage WO consists only of head and baseplate genes. The percentage of prophage WO genomes (N = 16) containing genes present in the active phage genome, WOCauB2, is depicted. Percentages were calculated for both all WO genomes (black) and only WO genomes with tail genes (grey). A gene map of WOCauB2 is shown above the plot with colors corresponding to the labels in Figure 1.

Figures 1 and 2 show that WO is comprised of core genes that are present in nearly all WO types and dispensable genes that are only present in some prophage WO types. When comparing gene content between the prophages with and without tail genes, there is a demarcation in whether certain functional modules are preserved. Across all genomes, the baseplate and head modules span 15 genes and may comprise a single module based on gene orientation and the close proximity of reading frames. These modules are also present in nearly all WO genomes (Figure 2). Furthermore, an integrase gene is present in 14/16 WO genomes, but this gene is highly variable and groups into three major phylogenetic clusters (Figure S1). For example, the family of integrase found in WOCauB2 is only present in four other WO genomes. In contrast, the dispensable gene clusters chiefly include the replication and tail/virulence gene modules. However, when just considering the prophages with tail genes, the tail genes and putative virulence genes *VrlA*, *VrlC*, and patatin are present in 100% of these WO prophages (Figure 2), suggesting that these genes play a functional role in tailed WO or *Wolbachia* biology. Indeed, patatins were originally annotated as storage proteins in potatoes, but they also have the lipolytic activity of phospholipase, catalyzing the cleavage of fatty acids from membrane lipids. Such enzymatic activity would be especially useful for phage WO when entering or exiting a membrane-bound intracellular bacteria.

Notably, there are a few WO prophages that contain genes that are not present in the reference genome of WOCauB2. These ‘unique genes’ are summarized in Table S2 and encode conserved hypothetical proteins, an M1 lysozyme, an addiction module toxin, an RNA-directed DNA polymerase, a helicase of the SNF2 family, and a DNA methylase. The presence of an addition module toxin but not an antitoxin gene to rescue it is unexpected. Toxin-antitoxin loci are common in mobile elements of free-living bacteria and employed as post-segregational killers to spread the mobile genetic elements they are associated with. The observation of a toxin gene in prophage WO without an antitoxin complement may indicate that this toxin has evolved a new function in the intracellular niche, such as killing the host *Wolbachia* cell during lysis. The presence of a DNA methylase in WO is also interesting, as it is present in a high fraction of WO haplotypes (9/16). Methylases are common on bacteriophages and may modify the DNA such that it becomes resistant to bacterial restriction systems.

**Figure 3:**
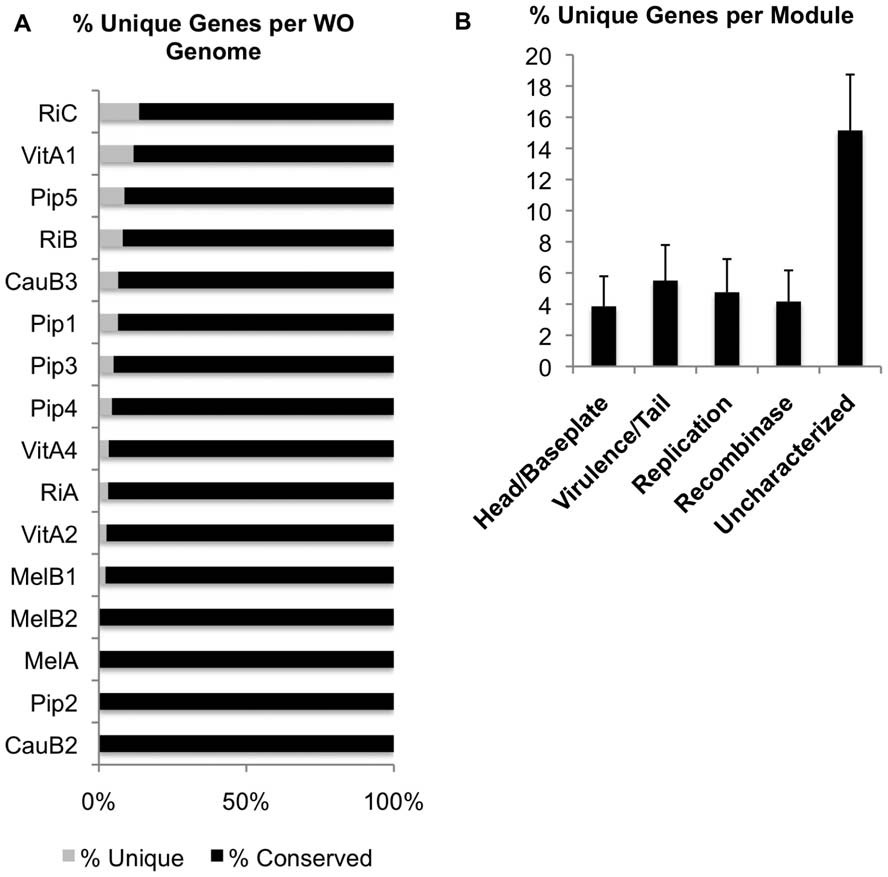
Unique genes in WO genomes are rare and scattered across functional modules. (A) The percentage of genes specific to a single prophage WO haplotype was calculated. (B) The percentage of unique genes present in each functional region across all WO prophages and standard error was identified by calculating the number of unique genes divided by the number of genes in the module.

Additionally, a few genes only occur once within the 16 WO genomes, making them unique to that particular prophage haplotype (Figure 3A, Table S3). These genes can comprise up to 13% of a prophage genome and are distributed broadly across 12/16 prophages with the exception of WOMelA, WOPip2, WOMelB2, and WOCauB2. Unique prophage genes can be classified into two groups – those that differentiate WO genomes but that also occur in other locations in the *Wolbachia* genome, including conserved hypothetical genes and a prophage uncharacterized gene (Table S3), and those genes that are found solely within the WO genome, including insecticidal toxin gene *SpvB* of WOCauB3, a bleomycin resistance gene found in WOPip4, ankyrin repeat protein-encoding genes, and conserved hypothetical protein-encoding genes.

As the prophage functional modules are comprised of operons that could be disrupted or enhanced by the acquisition of new gene content, we assessed if unique genes to a WO genome occurred in specific modules of the genome or randomly across the prophage genome. Novel genes are distributed in all prophage modules, with the highest percentage of novel genes found in the head/baseplate region (39.3%) and the virulence/tail region (25%). The remaining 10.7%, 7.1%, and 17.9% of unique genes are found in the replication, recombinase, and uncharacterized modules, respectively. The uncharacterized areas are found either between the head/baseplate module and the tail module (WOVitA1 and WOPip5) or the terminal region of the prophage upstream from the head genes (WOVitA4, WORiA, WOPip1, WOPip2, WOPip4, WOMelA, and WOMelB1). However, after normalizing the data to the gene number of these different regions (Figure 3B), as larger regions with more genes could contain more unique DNA, the fractions of unique genes per module were 3.9% (11 unique genes/285 total genes) for the head/baseplate module, 4.2% (2/48) for the recombinase region, 5.5% (7/127) for the virulence/tail module, 4.8% (3/63) for the replication module, and 15.2% (5/33) for the regions not assigned to a specific module. In summary, prophage WO is capable of acquiring a limited number of novel genes throughout the prophage WO genome, especially in the uncharacterized regions that may be under relaxed selection relative to the structural or lifecycle modules.

WO genomes are also clearly prone to degradation due to transposon insertions from multiple different families and gene mutations that lead to non-functional proteins (Figure S2A). One genome lacking a tail module, WORiC, and one genome for which the core modules are separated by a large genomic segment, WOMelB1, contain the highest fractions of pseudogenes (13.6% and 8.9%, respectively), including mutations to genes required to generate an active phage particle. The other five prophage genomes for which pseudogenes are present contain a lower percentage (2.1–.7.1%) of pseudogenes. After the data is normalized to account for the number of total genes in each module, transposons are also most frequent in the uncharacterized modular region (2/33 or 6.06%). Interestingly, there is little difference between degradation in prophages with and without tail genes, as 44% of pseudogenes are found in prophages with tail genes and 56% are found in prophages without tail genes (Fisher's exact test, two tailed, p = 0.7395).

### III. Is the WO integration site and mechanism conserved in *Wolbachia*?

To determine if the WO recombinases are homologous in *Wolbachia*, and thus mediate integration in a similar fashion, a protein alignment of the 14 site-specific recombinases was constructed. Three major families of integrases are represented in the WO prophages (Figure S1). First, the integrases encoded on a *Nasonia vitripennis w*VitA non-phage genome segment and WORiB are members of a family of phage-related tyrosine recombinases (93.9% amino acid identity) with the closest homolog found in *Ehrlichia canis*. Second, the integrases of WOPip2, WOPip3, WOPip4, WOVitA4, WOMelA and WOMelB are not closely related to the above integrases and belong to the serine recombinase family, and thus function using a different mechanism than the tyrosine recombinases [51]. These WO integrases share 84.4% amino acid identity. Finally, there are two more subgroups of recombinases including those in WOCauB2, WOCauB3, WOVitA2 and a WO remnant from *w*Ri (96.4% amino acid identity) and those in WOVitA1 and WORiC (83.3% amino acid identity). These two groups of integrases also belong to the serine recombinase family. The high level of genetic and functional diversity in the recombinase genes supports the lack of a common integration site for all WO haplotypes and could be an indication of mosaic evolution that appears to not extend to other prophage WO modules.

**Figure 4:**
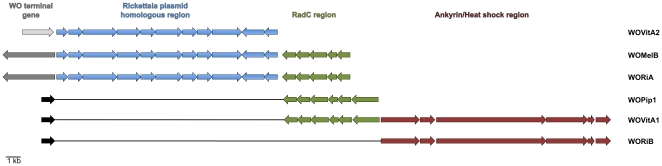
WO flanking regions contain conserved gene sets. The prophages WOVitA2, WOMelB, and WORiA are flanked on one end by a segment of genes that is conserved on a *Rickettsial* plasmid [36] (blue). In WOMelB and WORiA, a second conserved gene set (green), comprised of transcriptional regulators and the DNA repair gene *radC*, is found downstream of the *Rickettsia* gene homologs. This region is the prophage-flanking region in WOPip1 and WOVitA1. A third conserved gene segment (red) of ankyrin repeat proteins, a heat shock protein, and a conserved hypothetical protein, is found in WOVitA1 and flanking the prophage terminal gene in WORiB. Light gray: RepA; Dark gray: SNF2-family helicase; Black: patatin.

In order to confirm that members of prophage WO do not target conserved gene sequences for integration, the genes flanking prophage WO were compared. While there is no conserved gene set in all WO flanking regions, there are similarities between some WO types (Figure 4). Four prophages spanning three haplotypes (WOMelB, WORiA1, WORiA2, WOVitA2) have termini that are adjacent to a group of eleven genes also found in a plasmid from a *Rickettsia* symbiont of *Ixodes scapulari*s ticks [52]. The average pairwise nucleotide identity between these four prophages in this region is 85.3%. In all but WOVitA2, the gene preceding this cluster is an SNF2-family helicase that, in eukaryotes, can regulate transcription, maintain chromosome stability during mitosis, and process DNA damage [53]. The presence of these genes within a prophage region was first reported by Ishmael *et al.* [36], who also demonstrated by microarray analysis that three closely-related *Wolbachia* infections from fruit flies (*w*Au, *w*Sim, and *w*San) contained the same genetic region. The more divergent *Wolbachia* infections of *w*Pip and *w*Bm do not have this region. A BLASTx search determined that this region is found in the genomic shotgun sequences of *w*Wil and *w*Ana of *Drosophila*.

Immediately downstream from this conserved gene cluster in WOMelB and WORiA is a second set of conserved genes. These genes are also found adjacent to the phage terminal patatin gene in the phages WOPip1 and WOVitA1 (Figure 4), indicating a possible deletion of the *Rickettsia* homologs after prophage integration. These genes are oriented in the same direction, indicating that they may be cotranscribed, and include transcriptional regulators, the DNA repair protein RadC, and a conserved hypothetical protein. In WOVitA1 and WOPip1, this region extends to the DNA mismatch repair gene *mutL*. Interestingly, the region downstream of these genes in WOVitA1 (consisting of ankyrin repeat genes, the heat shock protein hspC2, and a hypothetical protein) is adjacent to the patatin in WORiB. This gene group is oriented in the same direction relative to each other, but opposite to the RadC gene cluster.

Eleven of the 16 prophages have the DNA repair protein RadC in the host *Wolbachia* regions that flank prophage WO, but never in the genome segment adjacent to the recombinase. The remaining WO phages have unknown flanking regions on the non-recombinase end (WOCauB2, WOPip2, WOPip3, and WOVitA4) or are not flanked by conserved gene segments (WORiC). While the majority of the prophages containing a *radC* homolog in the *Wolbachia* flanking regions do not have a large syntenous region, WOMelA, WOCauB3, WOPip4, and WOPip5 all contain a set of genes of similar function, including *radC*, transcriptional regulators, and hypothetical genes.

### IV. What is the relative strength of selection and recombination on phage WO protein evolution across the functional modules of the genome?

A complete view of prophage evolution in obligate intracellular bacteria involves a balance among the forces of genetic drift, adaptive evolution, functional constraints, and recombination. Analyses of molecular evolution, when applied to loci across the prophage modules, provide insight on how the modules may be differentially evolving. One important caution, however, is that temperate bacteriophages in general are highly recombinogenic [15]. There is abundant evidence of recombination within the minor capsid protein of WO [33], [45] and this raises a concern that different regions of the locus or genome may have experienced different evolutionary histories due to recombination; therefore the inference of selection using maximum likelihood phylogenetic approaches (i.e., PAML) is inappropriate. We analyzed variation in selection (ω = dN/dS) across the WO modules using the omegaMap software package [54]. This method employs a Bayesian approach to parameter estimation that is independent of phylogeny, and therefore, is less likely to falsely identify sites subject to diversifying selection in sequences that display clear evidence of recombination [55], [56].

To address how selection is affecting phage protein evolution, we applied the program omegaMap to test for variation in the nature and strength of selection (ω) across specific phage loci and whether this variation corresponds to specific phage functions. Strikingly, we found throughout the entire phage genome that the average ω value per gene was <1, indicating that WO prophage genes are overall under strong, purifying selection (Table 2). Individual residues rarely experience significant, positive selection. The only exception in the dataset is gp45, which is predicted to encode phospholipases of the patatin family that may facilitate phage entry or exit into or out of the *Wolbachia* cell by digesting lipids. Its four 3′ terminal nucleotides are under significant, positive selection (mean ω = 6.61–6.69; posterior probability of positive selection >0.95).

**Table 2:** Recombination and Selection in WO genes.

Gene	Length	n	θw	r2, d	|D′|, d	P_lk_	Pr2	P|D′|	2N_e_r/Locus (-InL)	2N_e_r/site/θw	Mean ω
**Untailed**											
**gp15**	222	6	0.105	−0.170	na	0.86	0.001	0.002	0(−13173.81)	0.000	0.111
**gp17**	957	6	0.119	−0.052	na	0	0.001	0	0(−304995.29)	0.000	0.179
**gp18**	366	6	0.111	0.177	na	0.004	0	0.051	0(−40621.8)	0.000	0.303
**gp19**	1002	6	0.069	−0.033	−0.028	0.372	0.014	0.012	0(−118253.22)	0.000	0.052
**gp21**	387	6	0.145	−0.129	−0.069	0	0	0	1.0(−70346.48)	0.018	0.277
**gp22**	462	6	0.154	−0.031	0.015	0.001	0.015	0.843	1.0(−95555.08)	0.014	0.206
**gp23**	438	6	0.119	−0.115	−0.085	0	0	0	1.0(−68581.6)	0.019	0.199
**Tailed**											
**gp15**	222	6	0.142	−0.077	−0.044	0	0.001	0.11	2.0(−22892.43)	0.063	0.090
**gp17**	957	6	0.131	−0.063	−0.073	0	0	0	2.0(−309686.81)	0.016	0.190
**gp18**	366	6	0.134	−0.148	−0.037	0	0	0.004	2.0(−50448.29)	0.041	0.286
**gp19**	1002	6	0.083	−0.036	−0.028	0	0	0	14.0(−154609.48)	0.167	0.056
**gp21**	387	6	0.165	−0.026	−0.004	0	0.029	0.414	1.0(−84186.15)	0.016	0.217
**gp22**	462	6	0.183	−0.081	−0.006	0	0	0.251	5.0(−117532.59)	0.059	0.173
**gp23**	438	6	0.214	−0.012	−0.015	0.002	0.091	0.028	3.0(−136640.64)	0.032	0.204
**gp28**	681	6	0.150	−0.143	−0.051	0	0	0	4.0(−210583.82)	0.039	0.155
**gp30**	1173	6	0.096	−0.076	0.015	0	0	0.978	11.0(−275129.17)	0.097	0.094
**gp31**	189	6	0.160	−0.198	−0.108	0	0	0	4.0(−17092.57)	0.132	0.171
**gp32**	1230	6	0.120	−0.228	−0.070	0	0	0	1.0(−534906.31)	0.007	0.274
**gp37**	495	6	0.143	−0.037	−0.041	0	0.006	0	2.0(−104015.99)	0.028	0.044
**gp40**	336	6	0.220	−0.007	0.003	0.636	0.254	0.645	0.0(−110168.19)	0.000	0.241
**gp41**	171	6	0.207	−0.065	−0.043	0.532	0.017	0.033	0.0(−18167.72)	0.000	0.201
**gp42**	867	6	0.196	−0.003	−0.005	0.999	0.157	0.089	0.0(−516916.65)	0.000	0.226
**gp45**	906	6	0.155	−0.155	−0.086	0	0	0	1.0(−393658.44)	0.007	0.236

θw – the population mutation rate per site; r – correlation coefficient between pairs of loci; r2, d – correlation of r^2^ with distance; D′|, d - correlation of |D′| with distance; P_lk_ , Pr2, P|D′| - probabilities resulting form testing the null hypothesis of no recombination with a likelihood permutation test; 2N_e_r/Locus (-InL) – the population recombination rate per locus under a coalescent framework; 2N_e_r/site/θw – the population recombination rate per locus per site; Mean ω-the mean ratio of dn/ds.

To statistically detect recombination within WO loci, we used the program LDhat [57], which analyzes sequence alignments and estimates the significance of intragenic recombination and the population rate of recombination (2*Ner*). It has been widely applied in several systems [57], including *Helicobacter pylori*, HIV, human mtDNA, and *Wolbachia* [45], [58]. Four estimates were calculated (Table 2) across genes that occurred in all prophages, or prophages that are separated into those with and without tail genes: (i) the population mutation rate (θw), (ii) the correlation coefficients of linkage disequilibrium (LD) with distance, (iii) the significance of the correlation using three different permutation tests, and (iv) the population recombination rate (2*N_e_r*) per locus under a coalescent framework. Genes used in the analysis are listed in Table S4 and Table S5 and were chosen based on the criteria that these genes lacked stop codon mutations. Sampling of the prophage taxa was restricted to the fully coding prophages with tail genes, WOCauB2, WOCauB3, WORiB, WOPip5, WOVitA1, and WOMelB, and the prophages without tail genes, WOPip1, WOPip2, WOPip3, WORiA, and, WOVitA4.

To determine if specific genes/modules are more likely to recombine than others, it is helpful to control for variation in population sizes (*N_e_*) that can affect the estimates of recombination rate. The ratio 2*N_e_r*/θ_W_ (per site) reduces to 2*N_e_r*/2*N_e_μ* and then to *r/μ*, yielding the likelihood of a base pair experiencing a recombination event relative to mutation in a given gene. The *r/μ* ratio for head and baseplate genes is notably different between the prophages with and without tail genes, where there is a significant eight-fold difference between the recombination rates (0.056, tailed vs 0.007, nontailed; MWU, two tailed p = 0.007) (Table 2). All seven genes analyzed from the head/baseplate modules for both the prophages with and without tail genes show evidence of recombination (at least 2/3 permutation tests with a p<0.05, Table 2). Seven of the nine genes in the virulence/tail module are positive for recombination; only the *tailU* (gp40) and late control D (gp42) genes do not show recombination (Table 2).

Among the prophages with tail genes, three genes in particular account for the higher rates of recombination relative to mutation in comparison to the prophages without tail genes: major capsid gene gp19 (0.167), putative virulence VrlC homolog gp30 (0.097), and conserved hypothetical gp31 (0.132) (Table 2). When just comparing the rate of recombination between modules within the prophages with tail genes, the tail module had the lowest rate of recombination (0.016), which is 3.6-fold lower than the head/baseplate module (0.056; MWU, two tailed, p = 0.01) and 3.4-fold lower than the virulence module (0.058; MWU, two tailed, p = 0.031).

**Figure 5:**
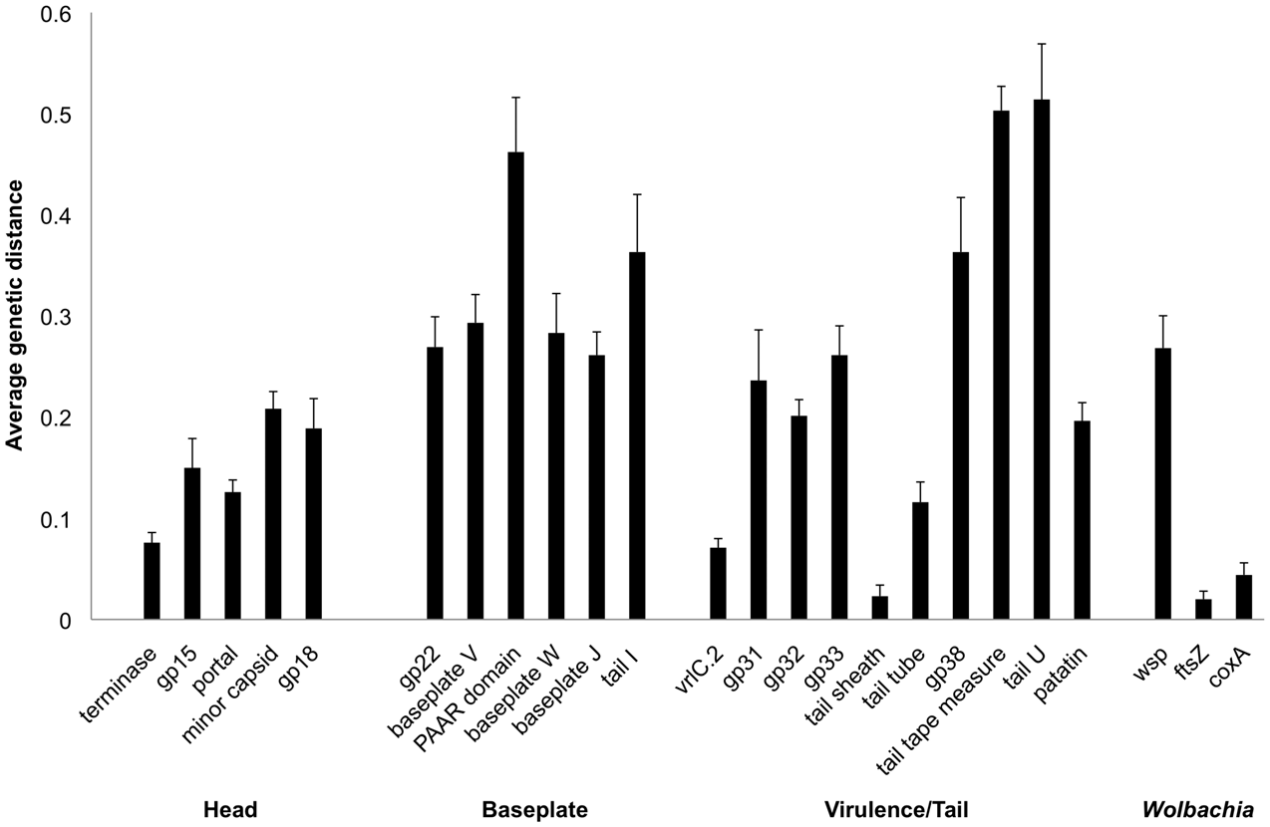
Mean genetic distance of WO and *Wolbachia* genes. The average genetic distance and standard error was calculated for genes across *Wolbachia* prophage WO haplotypes. The values for the hypervariable *Wolbachia* surface protein gene *wsp* and the highly conserved *Wolbachia* housekeeping genes *coxA* and *ftsZ* are provided for comparison. Black bars represent genes having a predicted function while gray bars represent genes for which no function can be predicted.

We also determined the average genetic distance, which is the average proportion of amino acid substitutions between a pair of proteins within a gene family, between homologs of genes across prophage WO (Figure 5). Proteins from the head region are the most evolutionarily conserved (average 0.1498) and have a significantly reduced genetic distance relative to the baseplate region (average 0.3218, MWU, two-tailed, p = 0.004) but not to the tail region (average 0.2484, MWU, two-tailed, p = 0.31). Elevated rates of change in the baseplate and some tail protein sequences is further evident by their similar genetic distances to the hypervariable *Wolbachia* surface protein *wsp*.

## Discussion

A null hypothesis for the evolution of dsDNA phages, known as the Modular Theory, is that phage genomes consist of clusters of functionally-related genes that can be interchanged by horizontal exchange within a large common gene pool [14], [15]. This theory of modular and mosaic evolution is well established in phage from free-living bacteria. However, access should not be uniform in the niche of an intracellular bacterium because the host cell is presumably a significant barrier to exchange with the global population of phages. In this regard, obligate intracellular bacteria are an ideal test of the Modular Theory as the intracellular niche may pose ecological restraints on exposure to novel phage gene pools.

In order to determine if Modular Theory applies to dsDNA phages in the obligate intracellular niche, we analyzed the prophage gene pool from *Wolbachia*. We show that WO prophages do not have a recent history of modular exchange and instead evolve through point mutations, deletion, recombination, and purifying selection. This non-mosaic evolution is also partly seen in the structural genes of the P2 family of phages that infect *E. coli* [59]. However, unlike P2 where evolution coincides with that of its host, phage WO evolution is incongruent from its host *Wolbachia* [45], a feature likely due to the rampant phage transfer between *Wolbachia* coinfections [28], [33], [34], [45].

While phage WO lacks mosaicism, it is modular in structure. The head and baseplate gene modules are found in every copy of WO, and the replication, virulence and tail modules are present in at least one WO type per *Wolbachia* genome. Given this pattern of one complete and at least one partial phage per *Wolbachia* genome, it is unsurprising that WO degradation is tolerated and pervasive. The presence of at least one intact copy of each known structural gene in a *Wolbachia* genome could allow for bacteriophage protein “commandeering” where the prophages that have mutations or lack the tail module could use proteins encoded on other WO prophages within the genome to complete their assembly and movement. A similar mechanism of transfer by defective phages has been shown for the Sp family from *E. coli* O157 [60].

Despite a reduced exposure to novel gene pools, WO is not completely immune to acquiring new genes. Transposons are frequently found within WO genomes and are known to play important roles in shaping the genomic diversity of *Wolbachia* [61], [62]. Additionally, the majority of WO prophages contain a few, unique genes with no defined role in phage functionality. Brussow and Hendrix [5] postulate that novel genes located within bacteriophages are frequently under their own transcriptional control and are maintained because they are advantageous to the bacterial host. Although a majority of novel WO genes are located in uncharacterized regions, some could be important to bacterial biology. Several of the novel genes are also found in non-phage regions of the *Wolbachia* genome, which indicates that WO could be exchanging new genes through recombination with the host genome.

Surprisingly, homologs of several genes required for complete function in dsDNA phages (holins, lysozymes, transcription factors) could not be identified in all prophage WO. It is possible that the large number of conserved hypothetical genes provide those missing functions. There is precedent for such genes that lack sequence homology to known functional phage proteins but perform identical roles [5], [63]. Further, the modular structure of phages is conserved among phages that infect Bacteria and Archaea, but there is little to no sequence homology between genes that provide an equivalent function.

Recombinases are one example of WO genes that are diverse in nucleotide identity but are functionally comparable. Prophage WO haplotypes encode a range of recombinases. Since recombinase genes are frequently interspersed throughout the *Wolbachia* genome, it is easy to extrapolate that recombination between WO and the host genome facilitates the switching of prophage recombinases. The diversity of recombinases in WO correlates with the apparent lack of a consensus in their integration site. Unlike prophages that recombine into a specific gene, such as a tRNA gene, the integration point of prophage WO cannot be predicted and seems to vary based on the prophage haplotype.

Evidence from ssDNA phages that do not evolve modularly has shown that structural genes (“self” genes) are more evolutionarily conserved, while genes that interact with the host (“nonself” genes) evolve more rapidly [64]. In this system, the viral coat proteins have fewer than 1% amino acid changes, while the assembly proteins have 1–10% amino acid differences, and the genes required for phage entry and release are more than 10% divergent. This theory offers one explanation for how phages isolated from diverse areas and at different times have a high degree of genetic similarity. The WO prophages follow this trend. The head module, which does not interact with the host, is the most conserved while the baseplate module, which is involved in phage-host recognition, is the most genetically divergent.

Temperate bacteriophages, such as WO, tend to be highly recombinogenic. Within WO, rates of intragenic recombination are 8-fold greater in genes from prophages with tail genes than in genes from prophages without tail genes. Since prophages with complete tail modules have a greater chance of forming virions and being transferred into new genomes, the increase in recombination could be due to a wider exposure over time to other WO phages. The question still remains if WO prophages that occur in the same genome can recombine with each other.

The nature of selection within prophage genes results in a complex dichotomy between what is advantageous for the bacteria versus what is best for the phage. Prophage WO genes are under strong purifying selection, where deleterious mutations are selected against and removed from the population. One major hypothesis of phage evolution is through illegitimate recombination, which often occurs within open reading frames [65]. If the recombination leads to knockdown of a functional module and lack of a viable phage, the deleterious event will be discarded [66], [67], [68]. In this case, the phage genes under strong purifying selection are akin to ‘housekeeping’ genes that are conserved to maintain function.

While phage WO is, to date, unique in the obligate intracellular bacteria, a modular dsDNA tailed phage, known as APSE, is present in the facultative symbiont of pea aphids, *Hamiltonella defensa* [69], [70], [71], [72]. Diversity in APSE is driven by recombination and it has >90% nucleotide identity with other APSE genomes [72], indicating that, like WO, it may not evolve by modular evolution. Other similarities between APSE and WO include the ability to gain novel genes and its variable copy number in host genomes [70]. If the Modular Theory does not apply to both WO and APSE, then it must be considered that phages in bacterial endosymbionts have a reduced ability to exchange DNA with other phages owing to their restricted niche and limited exposure to other phage gene pools.

## Materials and Methods

### Prophages used in this study

Prophages analyzed in this study were i) from whole genome *Wolbachia* sequences from the infections of *Culex pipiens* Pel (*w*Pip, NC_010981) [29], *Drosophila melanogaster* yw (*w*Mel, NC_002978) [30], *Drosophila simulans* Riverside (*w*Ri, NC_012416) [43], and ii) from shotgun or partial genome *Wolbachia* sequences from the infections of *Cadra cautella* (*w*CauB, AB161975.2, AB478515.1, AB478516.1) [44] and *Nasonia vitripennis* (*w*VitA, HQ906662, HQ906663, and HQ906664) [28]. Prophages were divided into head, baseplate, recombinase, replication, virulence, and tail regions based on the predicted function of groups of genes oriented in the same direction (Table 1). Functionality was inferred based on i) the current gene annotation found in NCBI, ii) the annotation of non-*Wolbachia* homologs identified in a tblastx search of the nr database, and/or iii) the presence of conserved protein domains.

### Identification of phage gene homologs and unique genes

A tblastx search using each annotated gene from the sixteen prophage WO genomes as the query was performed against the whole genome sequences of *w*Pip [29], *w*Mel [30], *w*Ri [43], and *w*VitA (unpublished data) and the prophage and flanking sequences of WOCauB2 and WOCauB3 [44]. Genes were considered homologs if there was greater than 50% amino acid homology over 30% of the coding length. The bacterial species from which the closest relatives were identified was noted. Genes that did not have a homolog in *Wolbachia*, and thus considered unique to their phage haplotype, were used as the query in a tblastx search against the NCBI nr database to identify potential homologs in other bacteria.

### Gene content and synteny

Prophage gene homologs of the WOCauB2 genes gp17, gp18, gp19, gp21, gp22, and gp23 (identified in the tBLASTx search described above) were aligned using the MUSCLE plugin [73] in Geneious version 5.0.4 [74]. Each representative from each haplotype was aligned with every other prophage WO homolog, and the percent nucleotide identity was compared between prophages integrated within the same *Wolbachia* genome and between prophages integrated in different *Wolbachia* genomes.

### Comparison of recombinases

The amino acid sequences of the annotated recombinases found in the WO prophages were aligned using MUSCLE. A neighbor-joining consensus phylogenetic tree using the Jukes-Cantor general distance model, no outgroup, and 100 bootstrap replicates was constructed using the Geneious Tree Builder. A blastx search of the nr database was performed to identify the closest homologs and recombinase protein families for each phage WO representative.

### Comparison of WO flanking regions

The *Wolbachia* genomic sequences flanking each prophage were compared using the Mauve [75], [76] plug-in in Geneious to identify homologous genes. For *w*CauB phages WOCauB2 and WOCauB3, the entire known flanking sequence was compared. For the phages for which the whole *Wolbachia* genome is sequenced, a minimum of 13.4 kb of flanking sequence was used for comparison.

### Recombination and selection

The prophage WO homologs of seven genes from the head/baseplate region (Table S4) and nine genes from the virulence/tail region (Table S5) were aligned using MUSCLE. Criteria for the taxa analyzed were (i) they must have coding genes and (ii) the taxa were consistent among all of the alignments. Analysis of the head/baseplate genes was restricted to WOCauB2, WOCauB3, WOPip1, WOPip2, WOPip3, WOPip5, WOMelB1, WOMelB2, WORiA, WORiB, WOVitA1 and WOVitA4. Analysis of the tail region was restricted to WOCauB2, WOCauB3, WOPip5, WOMelB1, WORiB, and WOVitA1. Indels were removed from the alignment using MacClade version 4.08 [77].

To investigate the influence of recombination, the program LDhat [57] was used. LDhat estimates the population recombination rate by composite-likelihood method and employs a parametric approach, based on natural coalescence, to estimate the scaled parameter 2*N*_e_ *r* where *N_e_* is the effective population size, and *r* is the rate of recombination. The estimate of the population recombination rate is normalized by the mutation rate (*θ*), which is estimated using a finite-series version of the Watterson estimator. All the data sets were run through both crossing-over model L and Gene conversion model C with respective gene length and average tract length of 50-bp and 100-bp for the analysis of biallelic sites. Because recombination tract lengths are unknown for *Wolbachia* and the estimates of 2*N*_e_ *r* can be highly dependent on the recombination tract lengths, recombination rates from the genetic exchange model producing the best-likelihood score are presented and should be interpreted with some caution. Nonetheless, all data sets produced the best-likelihood score with a gene conversion, 50-bp tract model.

In order to address the strength of adaptive evolution on point mutations, we used the method implemented in OmegaMap [54] that employs a Bayesian approach to parameter estimation that is independent of phylogeny, and therefore, less likely to falsely identify sites subject to diversifying selection in sequences that display clear evidence of recombination [55], [56]. The program estimates both variation in selection (*ω* = dn/ds) and the population recombination rate (*ρ*). The following prior distributions were used for the analyses: *μ*, *κ* and Ö_indel_: improper inverse, ù: inverse with range 0.01–10, *ρ*: inverse with range 0.01–10. The variable block model was chosen for both *ω* and *ρ*, with block sizes of 30 and 30, respectively. Analyses were performed with 500,000 iterations and 10 reorderings as suggested in omegaMap documentation (Wilson, 2006). To summarize the data, the Summarize module of omegaMap program, which summarizes the results from every 100^th^ generation of the run, was used. The first 50,000 sequences were discarded as a burn-in.

### Mean evolutionary distance

To estimate the level of conservation for individual proteins within the WO phage family, homologous amino acid sequences for each protein were first aligned using ClustalW2 (http://www.ebi.ac.uk/Tools/msa/clustalw2). The alignment was then imported into the program MEGA4 [78], and the overall mean distance was calculated under an equal input model. This model assumes that each amino acid site has the same substitution rate but adjusts for differing amino acid frequencies in the protein. Gaps within the alignment were ignored so that only sites that were present in all sequences were considered in the analysis. The overall mean distance represents the average proportion of amino acids that differ among the sequences aligned. For example, an overall mean distance of 0.2 indicates that, on average, the homologous proteins differ from one another in 20% of their amino acid residues. For comparison, the overall mean distances of two *Wolbachia* housekeeping genes, *ftsZ* and *coxA*, and one *Wolbachia* gene with a high level of variability among strains, *wsp*, were calculated using protein sequences from the same strains of *Wolbachia* in which the WO prophages are located. Standard error for each overall mean distance estimate was also calculated by MEGA4 using bootstrap analysis with 500 replications.

## Supporting Information

Figure S1 **WO Recombinases are Diverse.** A neighbor-joining phylogenetic tree based on nucleotide sequences demonstrates that prophage WO recombinases cluster into three major groups. Groups A belongs to the tyrosine-recombinase family. Groups B and C belong to the serine-recombinase family.
(TIF)

Figure S2 **Degradation of WO Genomes.** The number of transposon insertions and pseudogenes were tallied in order to measure the degradation and gene loss in WO prophage genomes. A) The fraction of transposase genes and pseudogenes out of the total number of genes in each prophage WO genomes are denoted along with the standard error of proportion. B) The fraction of transposons and pseudogenes per functional module, normalized to account for difference in module size, is shown.
(TIF)

Figure S3 **Synteny analysis of the WOCauB2 family of phages.** Alignments were performed between the prophages and flanking regions of WORiB, WOCauB2, and WOVitA2. Dotplot analysis shows that these prophage genomes are syntenous and contain few breakpoints between the genomes.
(TIF)

Table S1 **Average percent nucleotide identity for each prophage gene within a** ***Wolbachia*** **genome. Parentheses indicate the number of phage genes/haplotypes per** ***Wolbachia*** **.**
(DOC)

Table S2 **Genes present in WO haplotypes that are not present in WOCauB2.**
(DOC)

Table S3 **Genes found in a single WO haplotype.**
(DOC)

Table S4 **Genes used in selection and recombination analysis from the head and baseplate modules of both tailed and untailed phages.**
(DOC)

Table S5 **Genes used in selection and recombination analysis from the virulence and tail modules.**
(DOC)
