# Supplementary figures and images for: Evolutionary Genomics of a Temperate Bacteriophage in an Obligate Intracellular Bacteria (Wolbachia)
Source: PLoS One. 2011 Sep 14;6(9):e24984. doi: 10.1371/journal.pone.0024984 (PMC3173496; doi:10.1371/journal.pone.0024984)

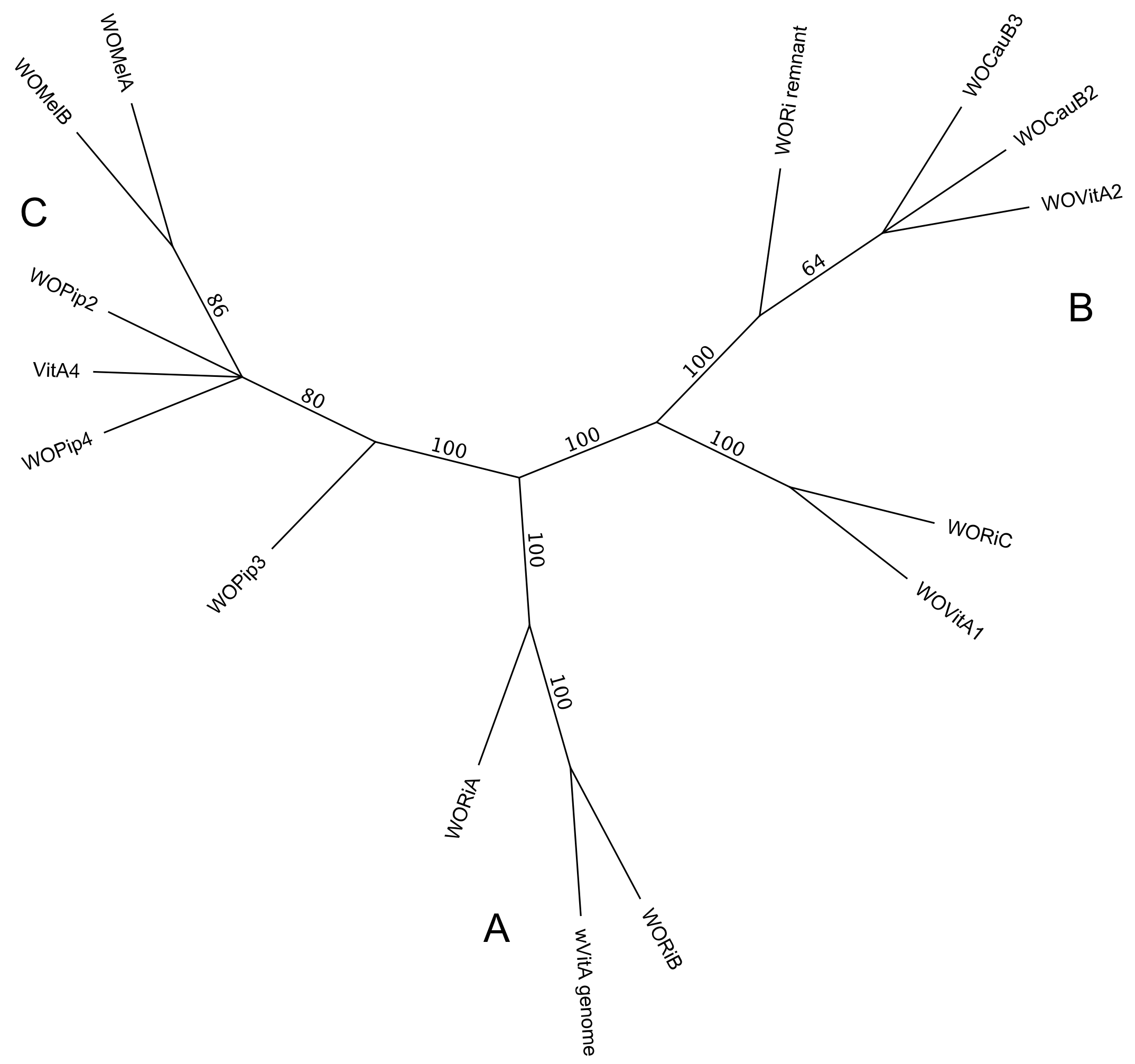

Supplement: Figure S1 — WO Recombinases are Diverse. A neighbor-joining phylogenetic tree based on nucleotide sequences demonstrates that prophage WO recombinases cluster into three major groups. Groups A belongs to the tyrosine-recombinase family. Groups B and C belong to the serine-recombinase family. (TIF) [file pone.0024984.s001.tif]

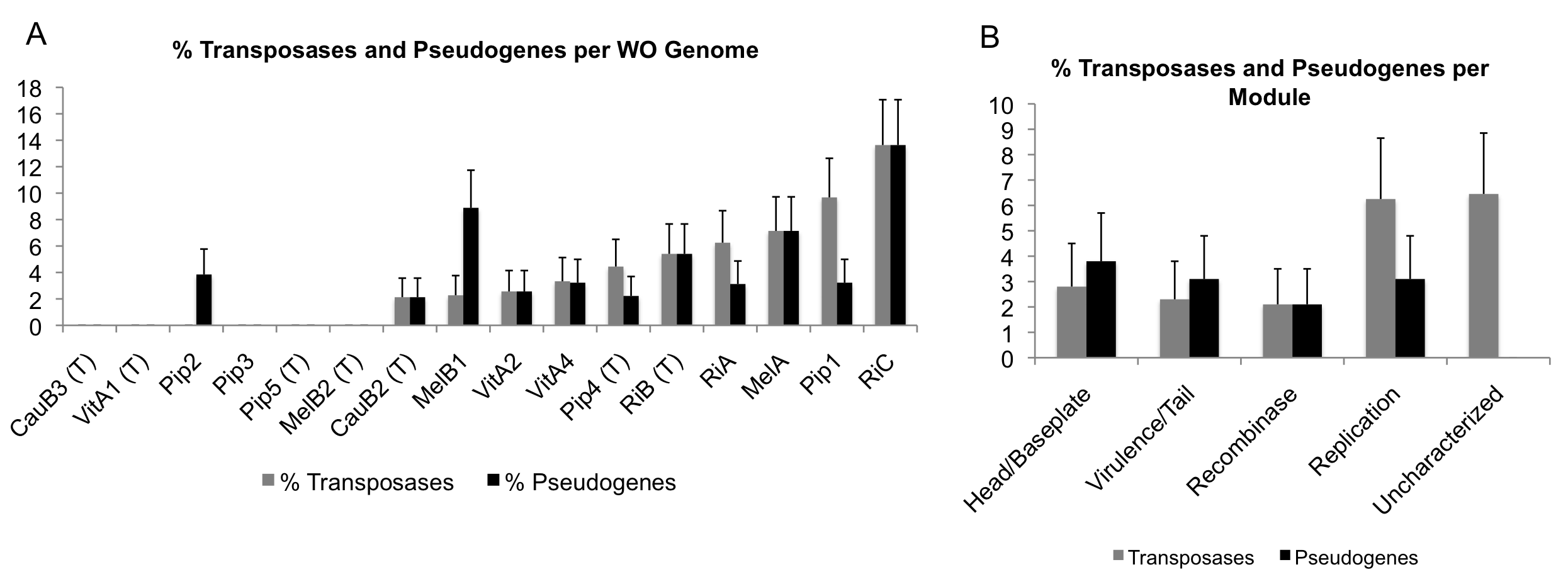

Supplement: Figure S2 — Degradation of WO Genomes. The number of transposon insertions and pseudogenes were tallied in order to measure the degradation and gene loss in WO prophage genomes. A) The fraction of transposase genes and pseudogenes out of the total number of genes in each prophage WO genomes are denoted along with the standard error of proportion. B) The fraction of transposons and pseudogenes per functional module, normalized to account for difference in module size, is shown. (TIF) [file pone.0024984.s002.tif]

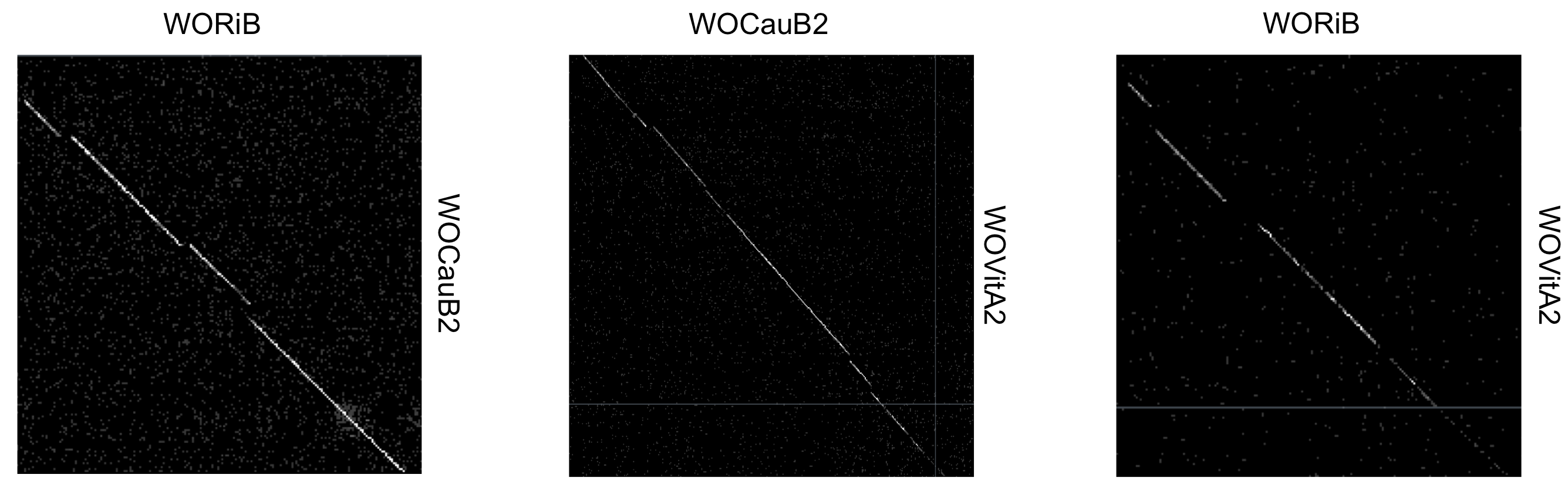

Supplement: Figure S3 — Synteny analysis of the WOCauB2 family of phages. Alignments were performed between the prophages and flanking regions of WORiB, WOCauB2, and WOVitA2. Dotplot analysis shows that these prophage genomes are syntenous and contain few breakpoints between the genomes. (TIF) [file pone.0024984.s003.tif]
