# Supplementary material for: Evolutionary Genomics of a Temperate Bacteriophage in an Obligate Intracellular Bacteria (Wolbachia)
Source: PLoS One. 2011 Sep 14;6(9):e24984. doi: 10.1371/journal.pone.0024984 (PMC3173496; doi:10.1371/journal.pone.0024984)
Supplement: Table S1 — Average percent nucleotide identity for each prophage gene within a Wolbachia genome. Parentheses indicate the number of phage genes/haplotypes per Wolbachia . (DOC) [file pone.0024984.s004.doc]

**Table S1**

|  | ***w*Pip (5)** | ***w*CauB (2)** | ***w*Vit (3)** | ***w*Ri (4)** | ***w*Mel (2)** |
| --- | --- | --- | --- | --- | --- |
| **gp15** | 99.1 | 76.9 | 83.1 | 82.7 | 76.5 |
| **gp17** | 85.2 | 83.9 | 79.3 | 83.0 | 75.7 |
| **gp18** | 89.8 | 81.3 | 87.4 | 84.0 | 79.8 |
| **gp19** | 96.2 | 94.8 | 93.0 | 89.3 | 84.0 |
| **gp21** | 85.1 | 95.2 | 85.9 | 63.5 | 65.7 |
| **gp22** | 84.4 | 76.8 | 80.3 | 77.8 | 68.8 |
| **gp23** | 88.7 | 53.4 | 85.9 | 79.5 | 78.7 |
| **Mean** | **89.8** | **80.3** | **85.0** | **80.0** | **75.6** |
