# Supplementary material for: Evolutionary Genomics of a Temperate Bacteriophage in an Obligate Intracellular Bacteria (Wolbachia)
Source: PLoS One. 2011 Sep 14;6(9):e24984. doi: 10.1371/journal.pone.0024984 (PMC3173496; doi:10.1371/journal.pone.0024984)
Supplement: Table S2 — Genes present in WO haplotypes that are not present in WOCauB2. (DOC) [file pone.0024984.s005.doc]

**Table S2:**

| **Gene product** | **Phage** | **Locus Tag** |
| --- | --- | --- |
| M1 lysozyme | WORiC | WRi_012650 |
|  | WOVitA4 | VA4gp17 |
| Addiction module toxin | WORiA | WRi_005580 |
|  |  | WRi_010240 |
|  | WOMelB1 | WRi_0600 |
|  | WOVitA2 | VA2gp17 |
| Hypothetical protein | WORiA | WRi_005590 |
|  |  | WRi_010250 |
|  | WOMelB1 | WD_0599 |
|  | WOVitA2 | VA2gp16 |
| Hypothetical protein | WORiA | WRi_010320 |
|  |  | WRi_005660 |
|  | WOMelA | WD_0259 |
|  | WOPip4 | WPa_0431 |
| RNA-directed DNA polymerase | WORiA | WRi_005670 |
|  |  | WRi_010330 |
|  | WOMelB1 | WD_0606 |
|  | WOMelA | WD_0258 |
| Hypothetical protein | WORiA | WRi_005680 |
|  |  | WRi_010340 |
|  | WOMelB1 | WD_0607 |
| Hypothetical protein | WORiA | WRi_005690 |
|  |  | WRi_010350 |
|  | WOMelB1 | WD_0608 |
| Helicase, SNF2-family | WORiA | WRi_005720 |
|  |  | WRi_010380 |
|  | WOMelB1 | WD_0610 |
|  | WOPip2 | WPa_0319 |
| Phage uncharacterized protein | WOPip5 | WPa_1305 |
|  | WORiB | WRi_007190 |
| DNA methylase | WORiA | WRi_005640 |
|  |  | WRi_010300 |
|  | WOMelA | WD_0263 |
|  | WOMelB1 | WD_0594 |
|  | WOPip1 | WPa_0258 |
|  | WOPip2 | WPa_0317 |
|  | WOPip4 | WPa_0429 |
|  | WOPip5 | WPa_1310 |
|  | WOVitA1 | VA1gp22 |
|  | WOVitA4 | VA4gp22 |
