# Supplementary material for: Evolutionary Genomics of a Temperate Bacteriophage in an Obligate Intracellular Bacteria (Wolbachia)
Source: PLoS One. 2011 Sep 14;6(9):e24984. doi: 10.1371/journal.pone.0024984 (PMC3173496; doi:10.1371/journal.pone.0024984)
Supplement: Table S3 — Genes found in a single WO haplotype. (DOC) [file pone.0024984.s006.doc]

**Table S3:**

| **Phage** | **Gene** | **Protein** | **Locus tag if present in *Wolbachia* genome** | **Closest bacterial non-*Wolbachia* relative** | **Organism** | **E Value** |
| --- | --- | --- | --- | --- | --- | --- |
| WOCauB3 | B3gp25 | CHP |  | PAAR-containing protein | *Aminobacterium colombiense* | 4.77E-18 |
| WOCauB3 | B3gp45 | SpvB |  | YD Repeat protein/ insecticidal toxin | *Desulfotomaculum acetoxidans* | 0 |
| WOCauB3 | B3gp46 | none |  | none |  |  |
| WOMelB1 | WD_0573 | CHP |  | none |  |  |
| WOPip1 | WPa_0260 | Phage protein |  | Prophage antirepressor | *Geobacter uraniireducens* | 1.92E-69 |
| WOPip1 | WPa_0261 | Phage ATPase |  | ATPase | *Rickettsia massiliae* | 1.20E-140 |
| WOPip3 | WPa_0337 | CHP | WD_0080 |  |  | 1.22E-55 |
| WOPip4 | WPa_0423 | Glycoxalase/ bleomycin resistance |  | Glycoxalase/ bleomycin resistance protein | *Janthinobacter sp.* | 1.97E-11 |
| WOPip4 | WPa_0428 | CHP | WD_0872 | none |  |  |
|  |  |  | WRi_008370 |  |  |  |
| WOPip5 | WPa_1295 | CHP |  | protein of unknown function | *Dyadobacter fermentans* | 1.30E-31 |
| WOPip5 | WPa_1313 | Phage protein |  | none |  |  |
| WOPip5 | WPa_1330 | Phage protein | WD_0404 | CHP | *Legionella pneumophila* | 6.17E-21 |
| WOPip5 | WPa_1332 | Phage protein |  | none |  |  |
| WORiA | WRi_005680 | CHP | WPa_0149 | none |  |  |
|  | WRi_010340 |  | WD_0607 |  |  |  |
| WORiB | WRi_006950 | CHP |  | none |  |  |
| WORiB | WRi_007190 | Phage protein | WPa_1361 | CHP | *Rickettsia belii* | 1.71E-119 |
|  |  |  | WD_0217 |  |  |  |
|  |  |  | WPa_1305 |  |  |  |
|  |  |  | WRi_p02070 |  |  |  |
| WORiB | WRi_007240 | Ankryin motif protein | WD_0753 | type III effector protein | *Ralstonia solancearum* | 3.51E-05 |
|  |  |  | WD_0754 |  |  |  |
| WORiC | WRi_012460 | Ankryin motif protein |  | none |  |  |
| WORiC | WRi_012580 | PQQ-repeat protein | WPa_0094 | Quinoprotein | *Magnetospirillum gryphiswaldense* | 5.70E-21 |
|  |  |  | WRi_007270 |  |  |  |
|  |  |  | WD_0750 |  |  |  |
| WORiC | WRi_012620 | CHP | WRi_013400 | ATPase | *Mucilaginibacter paludis* | 2.96E-113 |
| WOVitA1 | VA1gp24 | CHP |  | CHP | *Magnetococcus sp.* |  |
| WOVitA1 | VA1gp25 | Leucine-rich repeat protein | WD_0108 | none | *Naegleria gruberi* | 2.10E-08 |
|  |  |  | WPa_1086 |  |  |  |
| WOVitA1 | VA1gp26 | DNA polymerase III | WD_0108 | DNA pol III | *Oceanicaulis alexandrii* | 1.91E-14 |
|  |  |  | WPa_1086 |  |  |  |
| WOVitA1 | VA1gp27 | CHP |  | none |  |  |
| WOVitA1 | VA1gp28 | CHP |  | none |  |  |
| WOVitA1 | VA1gp48 | Ankryin motif protein |  | none |  |  |
| WOVitA4 | VA4gp18 | CHP |  | none |  |  |
