# Supplementary material for: Evolutionary Genomics of a Temperate Bacteriophage in an Obligate Intracellular Bacteria (Wolbachia)
Source: PLoS One. 2011 Sep 14;6(9):e24984. doi: 10.1371/journal.pone.0024984 (PMC3173496; doi:10.1371/journal.pone.0024984)
Supplement: Table S4 — Genes used in selection and recombination analysis from the head and baseplate modules of both tailed and untailed phages. (DOC) [file pone.0024984.s007.doc]

**Table S4:**

|  | **Gene** | | | | | | |
| --- | --- | --- | --- | --- | --- | --- | --- |
| **Prophage** | hypothetical | minor capsid | hypothetical | major capsid | tail Z | hypothetical | baseplate V |
| **WOCauB2** | **B2gp15** | **B2gp17** | **B2gp18** | **B2gp19** | **B2gp21** | **B2gp22** | **B2gp23** |
| WOCauB3 | B3gp16 | B3gp18 | B3gp19 | B3gp20 | B3gp22 | B3gp23 | B3gp24 |
| WOPip1 | WPa_0254 | WPa_0252 | WPa_0251 | WPa_0250 | WPa_0248 | WPa_0247 | WPa_0246 |
| WOPip2 | WPa_0313 | WPa_0311 | WPa_0310 | WPa_0309 | WPa_0307 | WPa_0306 | WPa_0305 |
| WOPip3 | WPa_0324 | WPa_0326 | WPa_0327 | WPa_0328 | WPa_0330 | WPa_0331 | WPa_0332 |
| WOPip5 | WPa_1306 | WPa_1303 | WPa_1302 | WPa_1301 | WPa_1299 | WPa_1298 | WPa_1297 |
| WOMelB1 | WD_0598 | WD_0602 | WD_0603 | WD_0604 | na | na | na |
| WOMelB2 | na | na | na | na | WD_0644 | WD_0643 | WD_0642 |
| WORiA-1 | WRi_005600 | wRi_005560 | wRi_005550 | wRi_005540 | wRi_005520 | wRi_005110 | wRi_005500 |
| WORiA-2 | WRi_010260 | wRi_010220 | wRi_010210 | wRi_010200 | wRi_010180 | wRi_010170 | wRi_010160 |
| WORiB | WRi_007200 | wRi_007170 | wRi_007160 | wRi_007150 | wRi_007130 | wRi_007120 | wRi_007110 |
| WOVitA1 | VA1gp17 | VA1gp15 | VA1gp14 | VA1gp13 | VA1gp11 | VA1gp10 | VA1gp9 |
| WOVitA4 | VA4gp19 | VA4gp15 | VA4gp14 | VA4gp13 | VA4gp11 | VA4gp10 | VA4g9 |
