# Supplementary material for: Evolutionary Genomics of a Temperate Bacteriophage in an Obligate Intracellular Bacteria (Wolbachia)
Source: PLoS One. 2011 Sep 14;6(9):e24984. doi: 10.1371/journal.pone.0024984 (PMC3173496; doi:10.1371/journal.pone.0024984)
Supplement: Table S5 — Genes used in selection and recombination analysis from the virulence and tail modules. (DOC) [file pone.0024984.s008.doc]

**Table S5**

|  | **Gene** | | | | | | | | |
| --- | --- | --- | --- | --- | --- | --- | --- | --- | --- |
| **Prophage** | hypothetical | vrlC | hypothetical | hypothetical | tail tube | tail U | tail X | late control D | patatin |
| **WOCauB2** | **B2gp28** | **B2gp30** | **B2gp31** | **B2gp32** | **B2gp37** | **B2gp40** | **B2gp41** | **B2gp42** | **B2gp45** |
| WOCauB3 | B3gp29 | B3gp31 | B3gp32 | B3gp33 | B3gp36 | B3gp39 | B3gp40 | B3gp41 | B3gp44 |
| WOPip5 | WPa_1321 | WPa_1323 | WPa_1324 | WPa_1325 | WPa_1328 | WPa_1334 | WPa_1335 | WPa_1336 | WPa_1340 |
| WOMelB1 | WD_0581 | WD_0579 | WD_0578 | WD_0577 | WD_0574 | WD_0569 | WD_0568 | WD_0567 | WD_0565 |
| WORiB | WRi_007060 | WRi_007020 | WRi_007010 | WRi_007000 | WRi_006970 | WRi_006930 | WRi_006920 | WRi_006910 | WRi_006880 |
| WOVitA1 | VA1gp35 | VA1gp37 | VA1gp38 | VA1gp39 | VA1gp41 | VA1gp45 | VA1gp46 | VA1gp47 | VA1gp51 |
